# Novel RNA Duplex Locks HIV-1 in a Latent State via Chromatin-mediated Transcriptional Silencing

**DOI:** 10.1038/mtna.2015.31

**Published:** 2015-10-27

**Authors:** Chantelle Ahlenstiel, Catalina Mendez, Steven T H Lim, Katherine Marks, Stuart Turville, David A Cooper, Anthony D Kelleher, Kazuo Suzuki

**Affiliations:** 1 The Kirby Institute, UNSW Australia, Sydney, New South Wales, Australia; 2 Immunovirology Laboratory, St. Vincent's Centre for Applied Medical Research, Darlinghurst, New South Wales, Australia

**Keywords:** epigenetics, heterochromatin, histone modifications, HIV-1, non-coding RNA, si/shRNA, transcriptional silencing, viral latency

## Abstract

Transcriptional gene silencing (TGS) of mammalian genes can be induced by short interfering RNA (siRNA) targeting promoter regions. We previously reported potent TGS of HIV-1 by siRNA (PromA), which targets tandem NF-κB motifs within the viral 5′LTR. In this study, we screened a siRNA panel with the aim of identifying novel 5′LTR targets, to provide multiplexing potential with enhanced viral silencing and application toward developing alternate therapeutic strategies. Systematic examination identified a novel siRNA target, si143, confirmed to induce TGS as the silencing mechanism. TGS was prolonged with virus suppression >12 days, despite a limited ability to induce post- TGS. Epigenetic changes associated with silencing were suggested by partial reversal by histone deacetylase inhibitors and confirmed by chromatin immunoprecipitation analyses, which showed induction of H3K27me3 and H3K9me3, reduction in H3K9Ac, and recruitment of argonaute-1, all characteristic marks of heterochromatin and TGS. Together, these epigenetic changes mimic those associated with HIV-1 latency. Further, robust resistance to reactivation was observed in the J-Lat 9.2 cell latency model, when transduced with shPromA and/or sh143. These data support si/shRNA-mediated TGS approaches to HIV-1 and provide alternate targets to pursue a functional cure, whereby the viral reservoir is locked in latency following antiretroviral therapy cessation.

## Introduction

The implementation of combined antiretroviral therapy (cART) has revolutionized HIV-1 treatment, transforming a previously universally progressively fatal disease into a manageable chronic infection. However, HIV-1–infected individuals experience an ongoing burden of lifelong treatment requiring compliance, despite long-term drug toxicities and increased morbidity and mortality. The current barrier to clearing HIV-1 infection is the reservoir, consisting of integrated virus in long-lived T cells and cells of myeloid lineage. It provides the source of viral recrudescence upon cessation of cART.^1^

Various approaches to overcome this barrier have been explored, including intensification of cART,^2,3,4,5,6,7^ very early cART intervention,^8^ and reservoir purging using viral reactivation strategies.^9,10,11,12,13^ However, these approaches have substantial limitations, primarily that upon cART cessation, there is rebound viraemia.^14^ Hence, we are exploring an alternative approach, whereby the reservoir is permanently locked in a silent latent state, allowing viral control in the absence of cART.

Our approach involves inducing and maintaining an HIV-1 latency-like state via short interfering (si) or short hairpin (sh) RNA-induced transcriptional gene silencing (TGS). These induce heterochromatin marks (histone methylation and histone deacetylation) via recruitment of histone methylases and deacetylases.^15,16^ We have developed an si/sh RNA, termed si/shPromA, which targets the highly conserved tandem NF-κB motifs in the HIV-1 5′LTR and induces prolonged virus suppression *in vitro*^17,18,19,20,21^ and *in vivo*.^22^ This is distinct from post-TGS (PTGS), which targets mRNA for degradation and usually results in rapid selection of escape mutants.^23^ Similar to our approach, several studies now describe TGS induction by certain siRNAs targeting around NF-κB–binding regions.^24,25,26^ A recent study describes a siRNA named “S4” targeting the triple motif NF-κB–binding region of HIV-1 subtype-C,^27^ which has a different NF-κB–binding sequence to our si/shPromA target. The S4-siRNA is reported to induce TGS associated with heterochromatin modification in the promoter region.^27^ In this study, we screened the HIV-1 5′LTR for novel candidate siRNA targets and investigated potential candidates for TGS induction comparable to our current lead candidate, PromA.

## Results

### A panel of siRNAs spans the HIV-1 5′LTR of NL4.3

**Figure 1:**
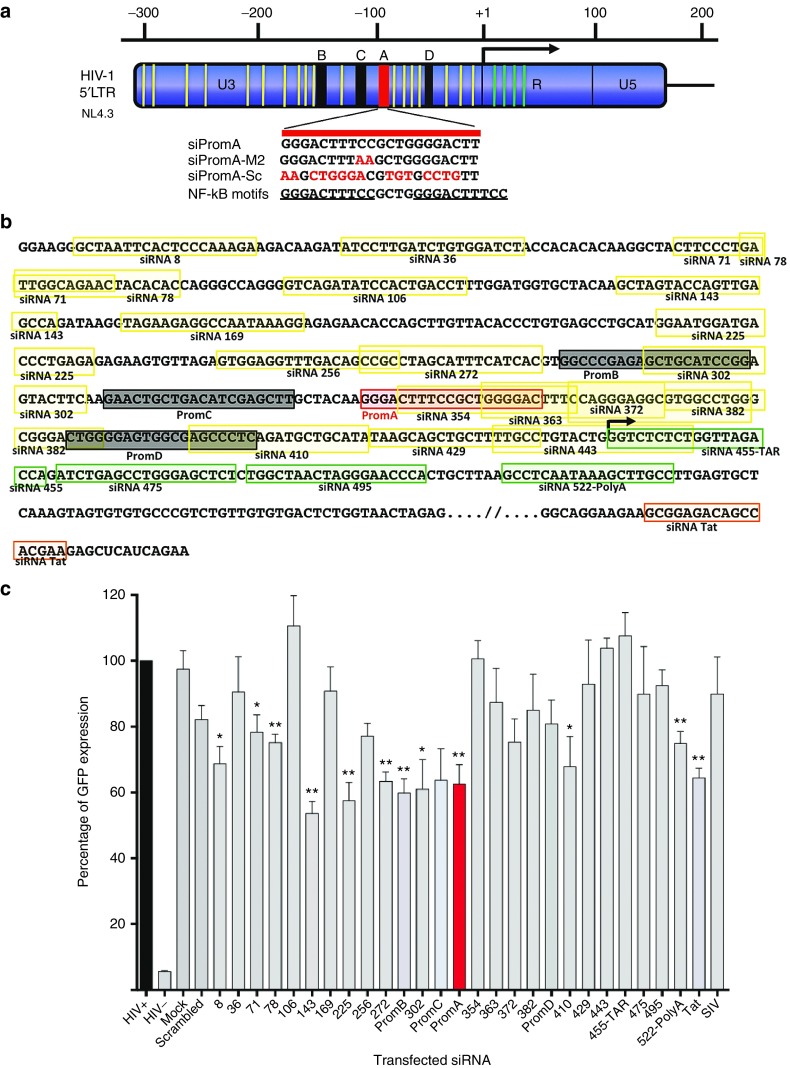
**Candidate TGS inducing siRNAs targeting the HIV-1 5′LTR region**. (**a**) Panel of siRNAs designed to target the HIV-1 5′LTR. Relative locations of candidate siRNAs are highlighted in yellow. Positions of previously reported siRNA, of which PromA (shown in red) targeting the NF-κB motifs is the most potent suppressor of HIV-1, while PromB, PromC, and PromD are shown in black. Candidate siRNAs designed to target the R region are shown in green. Sequence specificity controls, siPromA-M2 and siPromA-Sc, contain mismatched base pairs to siPromA. (**b**) Sequences of all siRNAs targeting the HIV-1 5′LTR region and part of the Tat exon. Bolded siRNA sequences boxed in yellow target the U3 region, while bolded siRNA sequence boxed in green and orange target the R region and Tat exon, respectively. PromA (bolded and boxed red), PromB, PromC, and PromD (bolded and boxed black) are shown for comparison. Nucleotide sequence shown is from HIV-1_NL4.3_. (**c**) Flow cytometry analysis of pseudotyped HIV-1 GFP expression in 293T cells transfected with a panel of candidate siRNAs targeting the HIV-1 5′LTR region. GFP expression was measured 48 hours posttransfection of siRNA. Data shown is mean ± SD from three independent experiments. *P ≤ 0.02, **P ≤ 0.008. The effect of the previously identified lead siRNA candidate for suppressing HIV-1 by TGS, PromA, is shown in red. Statistical comparisons were made between the HIV-1 GFP-pseudotyped positive control culture (black) and candidate siRNA-transfected cultures.

We previously demonstrated siRNA PromA targeting the HIV-1 5′LTR induces potent HIV-1 suppression, while other siRNAs induce variable levels of less sustained suppression (PromB), limited suppression (PromC), or no suppression (PromD)^19,20^ despite all four sequences being located within 90 bp of each other. To evaluate potential induction of viral suppression, we designed a comprehensive panel of 27 siRNAs spanning the HIV-1 5′LTR (**Figure 1a**,**b** and **Supplementary Table S1**). The panel was compiled according to the following criteria. Sequences were excluded if the target (i) included variable regions of the virus (designated as >10% sequence variation in the 2013 HIV sequence compendium alignment) or (ii) had homology with sequences in the human genome on BLAST search (defined as matching >17/19 bases). Sequences were not excluded if they (i) overlapped previously studied sequences, *e.g.*, PromA to PromD^19,20^ as off-setting of target sequences can alter efficacy substantially^28^ or (ii) were targets in DNA associated with nucleosomes, nuc-0 or nuc-1, despite these sequences being considered less accessible. To monitor artifactual responses, we included known siRNA sequence specificity controls for PromA, siPromA-M2 (mutated at positions 9 and 10) and a scrambled control, siPromA-Scram (containing the same GC content), and a control siRNA targeting simian immunodeficiency virus (**Figure 1a**).^18,19,20,29^

### Candidate siRNAs can induce HIV-1 suppression in a 293T pseudotyped virus system

The panel was screened for potential HIV-1 suppression using the VSV-G pseudotyped HIV-1-GFP reporter. Comparisons were made relative to the mock-transfected control (and not the scrambled siRNA control), as it was based on the PromA sequence and therefore does not reflect the GC content or the positioning of GCs in each of the siRNAs included in the screening panel. Flow cytometry analysis confirmed siRNAs PromA through PromD-mediated suppressive effects in this system, consistent with our previous studies^18,19,20,21^; siPromA and siPromB induced significantly lower GFP expression than siPromC and siPromD (both *P* = ≤ 0.008; **Figure 1c**). Screening the siRNA panel revealed that nine other candidates caused significant reduction in GFP expression; siRNAs 8, 71, (both *P* ≤ 0.02), 78, 143, 225, 272 (all *P* ≤ 0.008), 302, 410 (both *P* ≤ 0.02), and 522-PolyA (*P* ≤ 0.008). Importantly, siRNA targeting simian immunodeficiency virus, and scrambled siRNA controls did not show any significant decrease in GFP expression (**Figure 1c**). This procedure produced a number of candidate siRNAs with potential to suppress virus gene expression, via targets in the U3 region, including the HIV-1 5′LTR nuc-0 region.

### siRNAs targeting the U3 region can induce HIV-1 suppression in live virus strains

**Figure 2:**
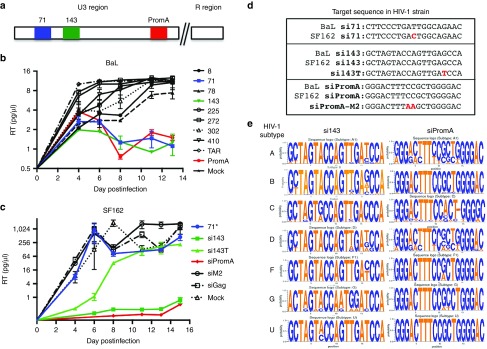
**Novel candidate siRNAs targeting the HIV-1 5**′**LTR region**. (**a**) The regions within HIV-1 5′LTR targeted by 71, 143, and PromA siRNAs are highlighted in blue, green, orange, and red, respectively. (**b**) Effect of siRNAs on the time course of HIV-1_BaL_ production in MAGIC-5 cells (HeLa cells stably transfected with CD4, CCR5, and CXCR4). (**c**) Effect of siRNAs on the time course of HIV-1_SF162_ production in HeLa T4+ cells. 5 × 10^4^ MAGIC-5 cells or HeLa-T4+ cells were transfected with 80 pmol/l of the appropriate siRNA, then infected using HIV-1_BaL_ (100 pg of RT/μl) or HIV-1_SF162_ (140 pg of RT/μl), respectively. Supernatants were harvested and virus production assessed using levels of RT activity. SiRNAs 71 (blue) and143 (dark green) profoundly suppressed HIV-1_BaL_ production for >12 days and at levels comparable to the current lead candidate, PromA (red), while only siRNA 143 (dark green) suppressed HIV-1_SF162_ production for >14 days to similar levels. Asterisk indicates a single mismatch in siRNA 71 target sequence of HIV-1_SF162_. (**d**) Subtype B HIV-1_BaL_ and HIV-1_SF162_ sequence alignment at targets of lead siRNA candidates. (**e**) Consensus sequence alignments of siRNAs 143 and PromA across HIV-1 subtypes A, B, C, D, F, G, and U. The 305 HIV-1 subtype sequences analyzed were obtained using QuickAlign Analysis from the Los Alamos National Laboratory HIV sequence database, which then generated the WebLogo 3 sequence logos^32,33^ and show both siRNAs target relatively conserved regions across HIV-1 subtypes. Gaps are indicated by dashes. The sequence logos display a graphical representation of the consensus sequence within a multiple sequence alignment of each HIV-1 subtype, with the stack height indicating sequence conservation at that position and the symbol height within the stack indicating relative nucleic acid frequency.^32,33^

To confirm our observations using replication-competent HIV-1, MAGIC-5 cells were infected with subtype B HIV-1_BaL_^30^ and reverse transcriptase (RT) activity measured over an extended infection time course. Two of the nine candidates, siRNAs 71 and 143, were located upstream of PromA (red box) (**Figure 2a**) and potently suppressed HIV-1_BaL_ to levels comparable to PromA (**Figure 2b**); ~12-fold reduction in RT activity compared to infected mock-transfected cells. Other candidates screened did not suppress HIV-1_BaL_ and were not investigated further.

The suppressive effect was confirmed using subtype B HIV-1_SF162_ infection (**Figure 2c**). Compared to HIV-1_BaL_, the sequenced HIV-1_SF162_ strain^31^ had one bp mismatch in the siRNA 71 target, while siRNA 143 and siPromA targets were identical (**Figure 2d**). To further confirm whether a single mismatch can diminish the suppressive effect, we included siRNA 143T, containing a single T mismatch as it corresponds to a common variation at position 16 across HIV-1 subtypes A, D, F, G, and U (**Figure 2e**) and the HIV-1_SF162_ 3′LTR GenBank sequence (accession number M65024.1). Both matched siRNAs, 143 and PromA, potently suppressed HIV-1_SF162_ productive infection with >1,000-fold reduction in RT activity compared to mock-transfected cells at day 15 postinfection. Interestingly, siRNA143T suppressed virus infection similarly to siRNA 143 and PromA up to day 6 postinfection but was unable to maintain virus suppression past this point. The siRNA containing an HIV-1_SF162_ mismatch, siRNA 71, showed no significant reduction in RT activity compared to infected mock-transfected cells. These data indicate that a single target mismatch is sufficient to disrupt prolonged siRNA-induced HIV-1 virus suppression. Because siRNAs 71 failed to suppress HIV-1_SF162_, siRNA 143 became the focus of investigation.

To determine sequence conservation of siRNA 143, we performed a sequence alignment across HIV-1 subtypes A through G and U and generated sequence logos (**Figure 2e**).^32,33^ SiRNA 143 sequence conservation showed 94.9% median identity over 19 nucleotide positions with an identity range of 9/19 nucleotides having >95% conservation, 13/19 nucleotides having >90% conservation, and 18/19 nucleotides having >80% conservation. As previously reported, siPromA is highly conserved across all subtypes, with the exception of a 1 bp deletion in subtype C and showed 98.4% median identity over 19 nucleotide positions (**Figure 2e**).

### Limited contribution by PTGS to the observed suppressive siRNA activity

**Figure 3:**
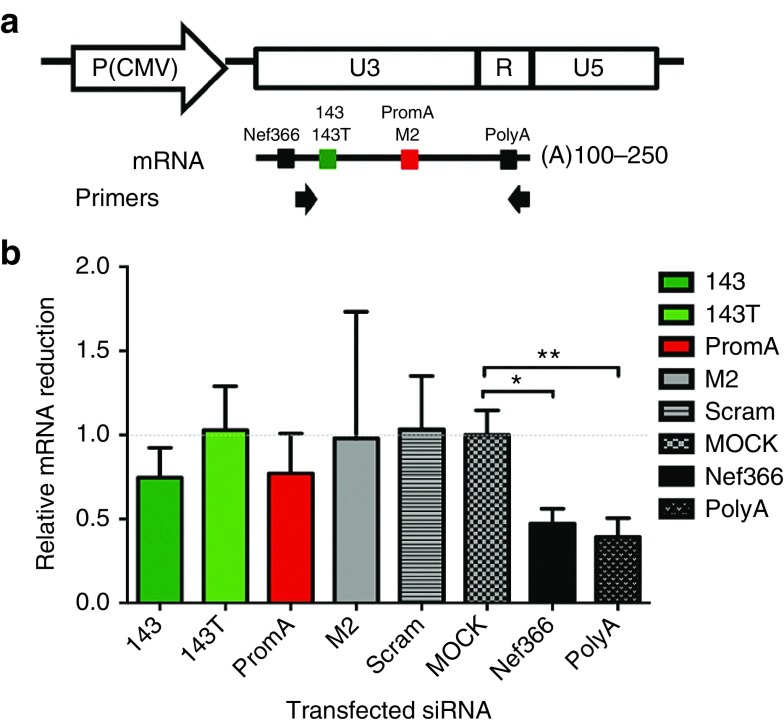
**SiRNAs targeting the U3 region of the HIV 5'LTR promoter have limited PTGS activity**. (**a**) Map of the HIV-1 3′LTR under control of the immediate early CMV promoter, with the location of sequences targeted by the selected candidate siRNAs (143/143T; green box, PromA; red box) and positive control siRNAs, PolyA and Nef366; black boxes). Arrows indicate the position of PCR primers used for the detection of HIV-LTR mRNA. (**b**) Assessment of the extent gene silencing contributed through PTGS following transfection of HeLa T4+ cells, stably expressing CMV-3LTR1-4, with the selected candidate siRNA panel. RT-PCR data are shown as a relative reduction in HIV-mRNA levels normalized to the mock transfection control. Data were normalized to a GAPDH control and statistical comparisons were made between the mock-transfected cultures and siRNA-transfected cultures. Data shown are from three independent experiments (mean ± SEM). *P = 0.028; **P = 0.009.

HIV-1 proviral DNA contains two identical LTR regions, the 5′LTR and 3′LTR. The 5′LTR functions as the promoter of the integrated viral genome, while the 3′LTR allows polyadenylation of nascent viral RNA and includes *nef* coding regions.^34^ Thus, PTGS could potentially contribute to the suppressive effects observed via siRNA targeting the 3′LTR. To investigate this, specifically at the mRNA level, we transfected a HeLa T4+ cell line stably expressing the 3′LTR sequence, designated CMV3′LTR1-4 (ref. 19), with candidate or appropriate control siRNAs (**Figure 3a**,**b**). Significant reductions in the HIV-1 3′LTR mRNA were found only with the positive control siRNAs, PolyA (*P* = 0.009) and Nef366 (*P* = 0.028), but not in any of the candidate siRNA-transfected cultures (**Figure 3b**), suggesting PTGS has limited contribution to the potent siRNA-induced HIV-1 suppression.

### Reactivation of HIV-1 transcription by treatment with HDAC inhibitors was observed in HIV-1 cultures suppressed by siRNA candidates

**Figure 4:**
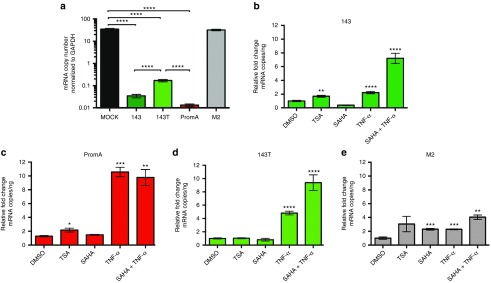
**Drug Reactivation of HIV-1 transcription was observed in HIV-1 cultures suppressed by siRNA candidates**. (**a**) Following HIV-1_SF162_ infection (140 pg of RT/μl) for 6 days and then transfection with 80 pmol/l siRNAs 143,143T, PromA, and PromA-M2, HIV infected DMSO control untreated cultures transfected with siRNA 143, and PromA showed suppressed levels of *gag* mRNA normalised to GAPDH expression compared to specificity control siRNAs 143T and siPromA-M2. Following established virus suppression for 24 hours with siRNAs (**b**) 143 (dark green), (**c**) PromA (red), (**d**) 143T (light green), and (**e**) M2 (gray), cultures were treated with TSA (50 nmol/l), SAHA (2.5 μmol/l), or TNF (10 ng/ml), or a combination of SAHA (2.5 μmol/l) and TNF (10 ng/ml) for 24 hours, then analyzed for intracellular viral RNA levels by RT-PCR. DMSO was added to untreated cultures at an equivalent concentration to drug treated cultures. Data are shown as a relative reduction in HIV-gag mRNA levels normalized to the HIV infected siRNA transfected DMSO control. Data shown are from three independent experiments (mean ± SEM). *P = 0.026, **P ≤ 0.002, ***P = 0.0002, ****P < 0.0001. All statistical analyses were performed using a Mann–Whitney test comparing the siRNA alone control with the drug activation cultures.

To further explore the mechanism responsible for siRNA 143-induced HIV-1 suppression, the effects of two histone deacetylase inhibitors (HDACi), which selectively inhibit type I and II HDACs, were assessed: trichostatin A (TSA),^35^ and vorinostat (or suberoylanilide hydroxamic acid (SAHA)). We previously demonstrated HDACi partially reverse the suppressive effects of promoter-targeted siRNA^19,20,21^ and hypothesized that siRNA 143 works by a similar epigenetic mechanism, partially reliant upon recruitment of HDACs to the 5′LTR region resulting in H3 deacetylation.^15,16^ We infected HeLa-T4+ cells with HIV-1_SF162_ and transfected the cultures with siRNAs 143, 143T, PromA, and PromA-M2 for 8 days. The siRNA-transfected cultures were treated with TSA, SAHA, or TNF, a potent latent HIV-1 reactivator,^16^ or combinations of these agents and intracellular viral mRNA levels were analyzed by RT-PCR. HIV-1_SF162_-infected cultures were significantly suppressed by siRNAs 143 and PromA (all *P* < 0.0001; **Figure 4a**), compared to their respective specificity controls (143T and PromA-M2) and the mock control, which all had at least 10-fold to 1,000-fold more virus mRNA expression (**Figure 4a**). HIV-infected cultures suppressed by siRNAs 143 or PromA transfection showed twofold increases in mRNA expression following TSA (TSA, 50 nmol/l) treatment (*P* ≤ 0.002 and *P* ≤ 0.03, respectively) indicating limited viral reactivation (**Figure 4b**,**c**). This treatment did not produce any significant increase in viral RNA expression in the mismatched siRNA 143T- or specificity control siRNA M2-transfected cultures above the high levels of viral production already present in these nonsuppressed cultures. Interestingly, SAHA (2.5 µmol/l) treatment did not increase mRNA expression in the HIV-1 suppressed siRNA 143- and PromA-transfected cultures (**Figure 4a**,**b**). Not surprisingly, TNF (10 ng/ml) significantly increased viral, cell-associated RNA levels across the entire siRNA panel, most markedly in siRNA PromA-treated cultures (11-fold; *P* ≤ 0.002; **Figure 4c**), 143T (5-fold, *P* < 0.0001; **Figure 4d**), PromA-M2 (~2-fold, *P* ≤ 0.002; **Figure 4e**) and 143 (~2-fold; *P* < 0.0001; **Figure 4b**). Combination treatment of SAHA and TNF resulted in increased HIV-1 mRNA levels, with significant increases observed in all siRNA transfected cultures; (143T 9-fold; *P* < 0.0001, 143 7-fold; *P* < 0.0001, and M2 4-fold; *P* ≤ 0.002) except PromA where the increase was comparable to TNF (PromA 10-fold; *P* ≤ 0.002; **Figure 4b**–**e**). Together, these data suggest silencing induced by siRNA 143, and PromA involves histone deacetylation via type I and/or type II HDACs.

### Heterochromatin markers were observed in HIV-1 cultures suppressed by novel siRNA candidate 143

**Figure 5:**
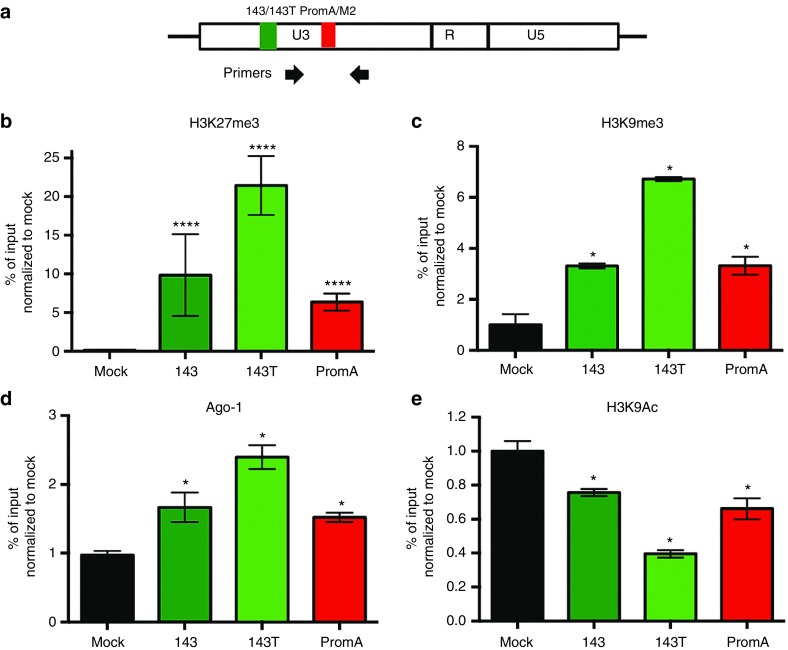
**Heterochromatin markers were assessed in candidate siRNA-transfected cultures**. (**a**) Arrows indicate the location of PCR primers used for SYBR-Green–based PCR amplification of DNA isolated by the ChIP process. Enrichment of heterochromatin markers (**b**) H3K27me3, (**c**) H3K9me3, and (**d**) Ago1 was found in siRNA 143, 143T, and PromA-transfected cultures compared to 48 hours mock-transfected cultures following 3 days of HIV-1_SF162_ infection. (**e**) Reduction of H3K9Ac expression was found in siRNA 143, 143T, and PromA-transfected cultures compared to mock-transfected cultures. Data shown are from three independent experiments (mean ± SEM). *P ≤ 0.05, ****P < 0.0001. Percentage of input was calculated, and data were normalized to the mock-transfected cultures for H3K27me3, H3K9me3, Ago1, and H3K9Ac.

To determine whether the siRNA 143-mediated HIV-1 suppression was associated with heterochromatin marks as described for PromA, we performed chromatin immunoprecipitation (ChIP) assays for epigenetic modifications in the 5′LTR 48 hours posttransfection. Primer location is shown in **Figure 5a**. We observed significant increases in H3K27me3; 10-fold in siRNA 143-transfected cultures (*P* <0.0001), and 22-fold in siRNA 143T and ~6.5-fold in PromA-transfected cultures (both *P* < 0.0001) compared with the mock-transfected cultures (**Figure 5b**) and significant increases in H3K9me3; >3-fold in siRNA 143- and PromA-transfected cultures and >7-fold in 143T-transfected cultures (all *P* < 0.05) (**Figure 5c**). Recruitment of Ago1 increased ~1.5-fold in siRNA 143- and PromA-transfected cultures, while increasing ~2.5-fold in siRNA 143T-transfected cultures (all *P* <0.05; **Figure 5d**). Significant reductions in the histone acetylation marker H3K9Ac were observed in siRNA 143-, 143T-, and PromA-transfected cultures (4-fold, 10-fold, and 6-fold, all *P* < 0.05; **Figure 5e**). Together, these data strongly suggest that the siRNA 143 mimetic silences HIV through TGS mechanisms.

### Novel candidate siRNAs targeting the HIV-1 5′LTR do not induce off-target effects

**Figure 6:**
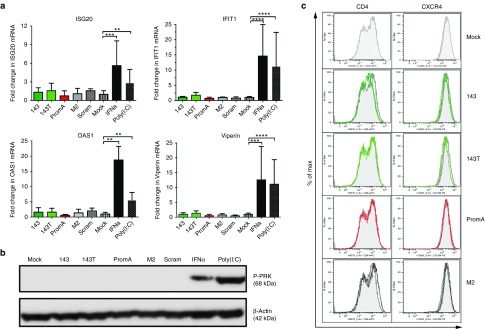
**SiRNA 143 mimetic targeting the HIV-1 5'LTR does not induce off target effects**. (**a**) HeLa T4+ cells were transfected with appropriate siRNAs for 48 hours. The mRNA expression levels of four IFNα response genes, ISG20, Viperin, IFIT1, and OAS1 were detected by SYBR-Green–based quantitative real-time PCR assays as described.^66^ ISG20, Viperin, IFIT1, and OAS1 were not elevated in cells transfected with candidate siRNAs. The fold change for each mRNA is shown. Mean ± SEM are plotted from triplicate experiments, and the data was normalized to the HeLa T4+ mock-transfected control. **P < 0.006, ***P = 0.003, ****P < 0.0001. (**b**) Activation of phosphorylated PKR was not detected in cells transfected with novel candidate siRNA for 48 hours. Phosphorylated dsRNA-dependent protein kinase R (P-PKR) was detected as described.^67^ Immunoblots were incubated with mouse anti-β-actin (A5441 clone AC-15, Sigma) or rabbit polyclonal antiphosphorylated PKR (pT451, BioSource, Invitrogen, Mulgrave, Australia), developed using Immuno-Star HRP chemiluminescent reagents (Bio-Rad) and visualized using a G:Box chemiluminescence imager and software (SynGene). β-Actin was used as a loading control. The positive controls were cells treated with IFNα (500 IU/ml) for 24 hours or cells treated with Poly(I:C) (10 ng/ml) for 6 h. (**c**) HeLa-T4+ cells transfected with candidate siRNA mimetics (143; dark green, 143T; light green, PromA; red and M2; black line overlay) display no change in expression of CD4 or CXCR4 when compared to mock-transfected cultures (gray area) at 48 hours posttransfection.

To determine whether the siRNA 143 mimetic has potential to mediate off-target effects, we analyzed four interferon-stimulated genes (ISGs) previously reported to detect IFNα responses induced by RNA duplexes,^36,37^ and the phosphorylation of PKR (P-PKR), following transfection of HeLa T4+ cells with candidate siRNAs for 48 hours. We observed no significant expression of ISG20, Viperin, OAS1 or IFIT1 in any siRNA-transfected cultures (**Figure 6a**), while cultures treated with IFNα or Poly (I:C) showed significantly increased expression of all four ISGs (all *P* < 0.05; **Figure 4a**). Additionally, there was no increase in PRK phosphorylation in any of the siRNA-transfected cultures (**Figure 6b**). We also assessed expression of HIV-1 CD4 receptor and CXCR4 coreceptor by flow cytometry, as downregulation may contribute to the silencing effect observed; however, no receptor downregulation was induced by any siRNAs at 48 hours posttransfection (**Figure 6c**). These data strongly suggest that there are no obvious off-target effects induced by the siRNA 143.

### Multiplexed approach, combining siPromA and si143, suppresses virus

**Figure 7:**
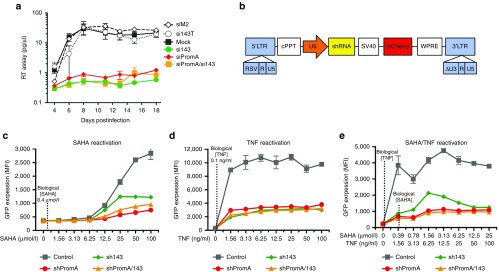
**Combining si/shRNAs PromA and 143 suppresses HIV in HeLa cells and diminishes reactivation in J-Lat 9.2 cells**. (**a**) HeLa T4+ cells were infected with HIV-1_SF162_, then co-transfected with siRNAs PromA and 143 or single siRNA treatments. RT activity was measured over an 18-day time course. (**b**) Structure of self-inactivating (SIN) lentivirus vector. SIN vector contains central polypurine tract (cPPT), U6 promoter (U6P), short hairpin RNA (shRNA), Simian virus 40 promoter (SV40), and mCherry reporter gene (mCherry). The mutant woodchuck promoter response element (WPREmt) and modified LTR, allow integration but not expression of viral genome. We utilized U6 promoter-driven shRNA PromA, 143 and control expression SIN lentivirus vectors with a mCherry reporter. (**c**) Reactivation of J-Lat 9.2 cells stably transduced with lentivirus vectors carrying shPromA, sh143, shPromA, and sh143 lentivirus vectors or control, treated with SAHA at various concentrations for 48 hours, show increased GFP expression upon activation. (**d**) Reactivation of J-Lat 9.2 cells stably transduced with lentivirus vectors carrying shPromA, sh143, shPromA, and sh143 lentivirus vectors or control, treated with TNF at various concentrations for 48 hours, show increased GFP expression upon activation. (**e**) Reactivation of J-Lat 9.2 cells stably transduced with lentivirus vectors carrying shPromA, sh143, shPromA, and sh143 lentivirus vectors or control, treated with a combination of SAHA and TNF at various concentrations for 48 hours, showed increased GFP expression upon activation.

To investigate whether combining siRNAs, PromA, and 143, in a multiplexed approach, would generate stronger induction of HIV-1 TGS, we performed single and double transfections of both siRNAs in HeLa T4+ cultures, then infected with HIV-1_SF162_ and measured RT activity over 18 days postinfection. We observed that the dual transfected cultures showed similar virus suppression overall compared to single transfections, with perhaps slightly more suppression at some time points (*e.g.*, day 10; **Figure 7a**). This data indicates that a multiplexed approach is at least as effective as single siRNAs at inducing virus suppression.

### shRNA targeting the 5′LTR diminishes reactivation induced by drug treatments in latent HIV-1 J-Lat 9.2 cells

To extend our study and investigate the effect of the siRNA143 target sequence in an HIV-1 latency model, we generated J-Lat 9.2 cells stably transduced with short hairpin (sh)143, shPromA or transduced with both shPromA and sh143 using separate lentivirus vectors (**Figure 7b**). We then attempted to reactivate integrated latent HIV-1 in J-Lat 9.2 cells using SAHA, TNF, or a combination of SAHA and TNF at various concentrations and measured GFP expression as a read out of HIV transcription, at 48 hours. We observed that J-Lat 9.2 cells transduced with sh143, shPromA, or dual sh143/shPromA were largely resistant to reactivation from SAHA (**Figure 7c**), TNF (**Figure 7d**), or combinations of SAHA/TNF (**Figure 7e**) at physiological concentrations^38,39^ and showed low level reactivation even at supraphysiological drug concentrations, while those transduced with a shRNA control showed highly elevated GFP expression, indicating reactivation of latent HIV-1 infection. Importantly, the dual sh143/shPromA transduced J-Lat 9.2 cell line appeared to show an additive effect in protecting against reactivation stimuli (**Figure 7e**). These data demonstrate that the HIV-1 latency model J-Lat 9.2 cells are largely resistant to reactivation by drug treatments when transduced with sh143 and/or shPromA.

### shPromA-transduced monocyte-derived macrophages can suppress HIV-1_JRFL_ infection

**Figure 8:**
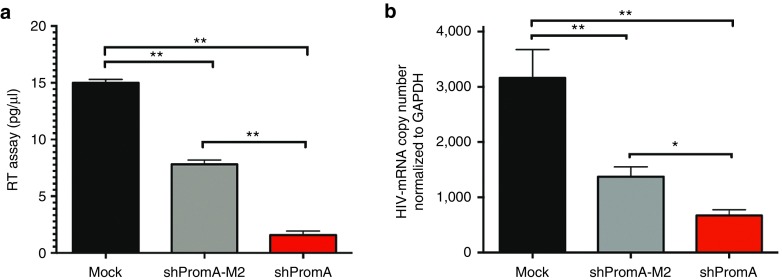
**shPromA induces potent HIV-1 suppression in monocyte-derived macrophages**. Monocyte derived macrophages (MDMs) were purified from PBMCs and differentiated for 7 days prior to being transduced with lentiviral vector expressing shPromA (red bar), shPromA-M2 (gray bar) or mock-transduced (black bar). MDM cultures were transduced for 5 days prior to infection with HIV-1_JRFL_ for 8 days. (**a**) RT activity was measured 8 days postinfection and showed potent virus suppression in shPromA-transduced MDM cultures. (**b**) HIV-1 mRNA levels were analyzed 8 days postinfection and also showed potent virus suppression in shPromA-transduced MDM cultures. **P ≤ 0.02, *P = 0.05.

The latent HIV-1 reservoir predominantly resides in long-lived T cells and cells of myeloid lineage. To investigate the observed suppressive effect of siRNA promoter targets on HIV-1 in primary cells, we extended our investigation to include monocyte-derived macrophages (MDMs). MDMs prepared from PMBCs were cultured for 7 days, and then transduced with lentivirus shRNA PromA (shPromA) for 5 days, followed by infection with HIV-1_JFRL_ strain, and analysis of RT activity and mRNA levels was performed at 8 days postinfection. Transduction efficiency of MDMs was ~20% as measured by GFP expression. Suppression of HIV-1_JRFL_ was observed in shPromA-transduced cultures, with 3.3-fold and 5-fold decreases in RT activity compared to shPromA-M2 and Mock control cultures, respectively (**Figure 8a**; both *P* = 0.02). Similarly, reductions in HIV-1 mRNA levels were observed in shPromA-transduced cultures, with ~1.3-fold and ~4-fold decreases observed compared to both shPromA-M2 and mock control cultures, respectively (**Figure 8b**; both *P* = 0.02). Considering the low transduction efficiency, these data demonstrate that promoter-targeted shRNA can potently suppress HIV-1 in primary MDM cultures.

## Discussion

Eradication of the HIV-1 latent reservoir presents a challenging barrier to advancing HIV therapeutics. Our study has approached this conundrum from the novel perspective of exploiting characteristics of HIV-1 latency by identifying siRNAs that mimic and reinforce these naturally occurring epigenetic modifications through induced TGS of the integrated provirus by targeting the 5′LTR. Our initial siRNA screen using HIV-1 pseudotyped virus and GFP reporter system identified nine novel siRNA candidates that demonstrated significant, but variable, degrees of suppression. Each candidate targeted sequences within the 5′LTR U3 region including those associated with nuc-0. The HIV-1 pseudotyped infection system provided a standardized, lower biocontainment, relatively rapid screening method. However, this single round infection does not replicate biological events of HIV-1 infection.

Upon further examination of the nine siRNA candidates identified by screening, only siRNA 143 demonstrated prolonged suppression of HIV-1 in both subtype B HIV-1_BaL_ and HIV-1_SF162_ strains to levels comparable to the previously identified siRNA PromA. This is likely due primarily to variations in sequence identity between the two strains. SiRNA 71 may suppress HIV-1_SF162_ if completely matched, although this needs to be confirmed experimentally. However, due to higher sequence variation in the targets of these two candidates, we focused on the potent suppressor siRNA143, which, like siRNA Prom A, showed a relatively conserved sequence identity across HIV-1 subtypes. However, a 1 bp deletion in subtype C may render siPromA less effective for this subtype. No deletions or insertions are present in the region targeted by siRNA 143 mimetic, which may offer additional protection against subtype C, which accounts for ~48% of all global infections.^40,41^ Multiplexing delivery of these siRNAs may allow exploitation of complementary efficacies.

Since siRNA 143 appeared to cause profound and prolonged viral suppression *in vitro*, the next step was to determine its mechanism of action. SiRNA 143 did not appear to induce effective PTGS at the mRNA level. PTGS functions through degradation of mRNA transcripts, is dependent on active transcription, and is thus prone to viral escape.^42^ A further major caveat of any PTGS-associated RNAi therapeutic is inability to degrade latent HIV-1 provirus due to the lack of active transcription. To circumvent this limitation, we focused on identifying siRNA mimetics capable of targeting both actively transcribing and latent provirus.

Although we found no significant induction of PTGS in the 3′LTR region using promoter-targeted siRNAs, another study has identified five cellular miRNAs enriched in latently infected patient resting CD4+ T cells, that specifically target the 3′LTR.^43^ The study hypothesizes that a panel of anti-miRNAs, including miR-28, miR-125b, miR-150, miR-223, and miR-382, could be combined as a miRNA inhibitor panel for use in activating latent HIV-1 to purge the reservoir.^43^ These miRNA sequences have the potential to target similar regions in the 5′LTR; however, this has not been investigated.

Given that there was little evidence of siRNA143 acting by PTGS and the time course of suppression was characteristic of siRNA-induced TGS, we sought evidence for this pathway, initially exploring the effects of HDAC inhibitors, TSA and SAHA, on the ability of siRNA 143 to maintain viral suppression. Consistent with the hypothesis that siRNA-induced TGS of HIV-1 is associated with epigenetic changes, treatment of siRNA 143- and PromA-transfected cultures with TSA (but not SAHA) showed significant increases of HIV-1 mRNA. This may be due to TSA having 30-fold stronger HDAC type I/II inhibitory activity in comparison to SAHA.^44,45^ We were somewhat surprised that SAHA treatment did not result in increased virus reactivation in either siRNA 143 or PromA, even though this HDAC inhibitor was active as indicated by increased mRNA levels in the combined SAHA+TNF treatments compared to individual drug treatments. Thus, the relative abilities of these two HDACi (TSA and SAHA) to inhibit specific HDAC isoenzymes, both in terms of specificity and potency, likely explains the differences seen in virus reactivation and may depend on the specific HDACs recruited. The partial reactivation we observed in siRNA cultures treated with TSA and SAHA, which was far less than TNF induced reactivation, is indicative that factors in addition to HDACs, such as recruitment of histone methytransferases, are involved in the induction and maintenance of siRNA-induced TGS and HDACi impact on only one part of this complex process. The dramatic effect of TNF treatment on all siRNA-treated cultures was expected, particularly in siPromA-treated cultures, due to siPromA targeting the NF-κB motifs in the 5′LTR and TNF being a potent inducer of NF-κB. Also as expected, the specificity controls, siRNA 143T and siPromA-M2, did not show any significant increase in virus reactivation with TSA treatment, since these are both inactive mutants that do not induce virus suppression.

Consistent with the HDACi effects on siRNA 143-induced viral silencing, ChIP assays showed significantly increased levels of histone methylation (H3K27me3 and H3K9me3) and decreased histone deacetylation (H3K9Ac) in 5′LTR-associated chromatin. These changes are consistent with induction of heterochromatin in this region of the integrated virus. These changes are also consistent with previously reported changes induced by siRNA PromA,^18,19,21^ which were recapitulated in data presented here. Importantly, these induced epigenetic changes are similar to those seen in latent forms of HIV-1.^15,16^

Recruitment of Ago1, an essential component of the RITS complex that drives TGS, was a feature induced by siRNA 143 and strongly supports the silencing process is similar to that induced by siRNA PromA. The mutant specificity control, siRNA 143T, induced a similar epigenetic profile compared to siRNAs 143 and PromA 2 days posttransfection, suggesting that although TGS induction was similar, the nonsustained suppression observed at day 6 postinfection (**Figure 2c**) indicates that the mutant was less efficient at maintaining epigenetic changes. These data suggest that induction and maintenance of epigenetic changes are related but separate dynamic processes. Continuous siRNA delivery, such as retroviral shRNA delivery, is likely to bolster the maintenance of this process.^46^

**Figure 9:**
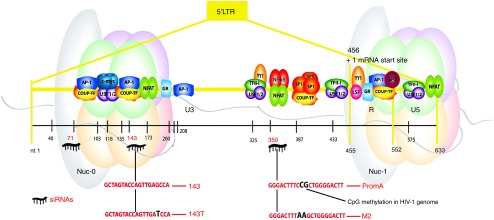
**Schematic model of transcription factors and targeted sites of siRNA mimetics 143 and PromA in the HIV-1 5′LTR**. Nucleosomes are comprised of histone 1 linker (gray) and four histone pairs (shown in green, orange, purple, and pink) that generate the histone octamer. Activator protein 1 (AP-1), COUP-TF, upstream stimulatory factor 1/2 (USF 1/2), nuclear factor of activated T cells (NFAT), glucocorticoid receptor (GR), transcription factor II-I (TF II-I), yin yang 1 (YY1), NF-κB, specificity protein 1 (SP1), late SV40 factor (LSF), specificity protein 3 (SP3).

TNF potently activates HIV transcription, particularly through NF-κB–mediated pathways by increasing expression of several host transcription factors that act directly on the HIV-1 promoter^16^ and therefore potently challenges siRNA-induced proviral silencing. Differential responses to TNF in cultures silenced by siRNAs-inducing TGS demonstrated that while TNF treatment showed significant increase in HIV-1 mRNA across all siRNA cultures, it was particularly efficient in reactivating PromA-suppressed cultures. This is likely related to siRNA PromA targeting NF-κB binding sites. In contrast, siRNA 143 targets a region ~200 bp upstream which lies directly within the nuc-0 region^47^ and has multiple host transcription factor–binding sites in the surrounding sequences (**Figure 9**). This is in stark contrast to siRNA PromA, which is located directly between nuc-0 and nuc-1, at least 100 bp from each nucleosome (**Figure 9**). It is tempting to speculate that one potential mechanism of HIV-1 suppression mediated by the siRNA 143 mimetic is due to disruption of one the protranscriptional effects of host transcription factors (*e.g.*, AP-1, NFAT), which are activated by TNF through different pathways.^48,49^ Interestingly, the mutant specificity control, siRNA 143T, demonstrated an increased response to TNF compared to siRNA 143 (~2-fold increase). This is consistent with the single-nucleotide mismatch weakening the silencing effect, thereby increasing susceptibility to reactivation by both HDACi and TNF.

Jurkat latent (J-Lat) cell lines have been extensively used in HIV latency studies, where DNA methylation and epigenetic factors have been implicated in silencing.^50,51^ There are several advantages of using this latency model. These include (i) that cells carry a known virus sequence,^52^ (ii) there are no “dead viruses” to potentially contribute noise to ChIP results, (iii) latency was a spontaneous event occurring during an active infection, (iv) no virions are produced from these cells, (v) the provirus is inducible, meaning the cells are carrying true latent proviruses, and (vi) expression of GFP protein is driven from activation of viral 5′ LTR promoter, which avoids the caveats of measuring read-through transcription as viral reactivation. Following an analysis of three J-Lat clones, 3.2, 8.2, and 9.2, we selected J-Lat clone 9.2 due to undetectable background GFP expression, reactivation with a wide range of stimulus and the expression of viral Gag and ^p55^Gag precursor proteins during viral reactivation.^52^ Additionally, HIV latency in the J-Lat 9.2 cell line is reported to occur via epigenetic modifications, specifically cytosine methylation, of two provirus CpG islands located in nucleosome-free regions that flank the transcription start site (*i.e.*, between nuc-0/nuc-1 and nuc-1/nuc-2).^50^ The nucleosome-free region between nuc-0 and -1 is one area rich in transcription factors and includes the siRNA target site for PromA, while the 143 target site is contained within nuc-0 and also has multiple transcription factor binding sites (**Figure 9**).

Therefore, the J-Lat 9.2 cell model of HIV-1 latency provided an ideal system in which to assess the effect of continuous siRNA delivery and its impact on reactivation of latent HIV-1. We generated transduced J-Lat 9.2 cell lines stably expressing sh143, shPromA, or both sh143/shPromA and exposed the cells to various reactivation stimuli. Cells transduced with sh143 and/or shPromA clearly demonstrated robust resistance to reactivation following stimulation with SAHA and/or TNF compared to parental J-Lat 9.2 control cells at physiological concentrations,^38,39^ although low-level reactivation was observed at supra-physiological concentrations (**Figure 7**). These findings are important in the context of gene therapy applications, where patients could be exposed to various immune and inflammatory stimuli. Additionally, “kick and kill” approaches have shown reactivation of latent provirus in resting CD4+ T cells derived from HIV-1 positive individuals occurs at the pharmacologically relevant concentration of 335 nmol/l SAHA (vorinostat),^10^ without reducing the HIV reservoir.^38^ In contrast, our findings clearly demonstrate robust resistance to SAHA reactivation in sh143 and/or shPromA transduced J-Lat 9.2 latent cell lines at this concentration.

The location of the siRNA143 target sequence within nuc-0–associated viral DNA is somewhat counterintuitive, as this type of region is considered less accessible and therefore less likely to be a suitable target. Another difference is siRNA 143 lacks a CpG site. CpG methylation is known to stabilize silencing of genes and is important for reinforcement of HIV-1 latency.^53^ By contrast, siRNA PromA includes a CpG site that is methylated after silencing.^20^ These observations challenge preconceived ideas regarding design of TGS-inducing siRNAs and are indicative of the paucity of understanding basic mechanisms underlying mammalian TGS. Importantly, unlike previous reports,^37,54,55,56,57,58,59,60^ no off-target effects can be attributed to the observed silencing effect of mimetic siRNA 143 through changes in ISG expression, P-PKR or HIV-1 CD4 receptor or CXCR4 co-receptor.

The potent HIV-1_JRFL_ suppression observed in shPromA-transduced MDM cultures suggests that this approach to controlling the HIV-1 reservoir is applicable across a range of cell types and viral integration sites. This is also supported by our *in vivo* data,^22^ and we plan to extend these studies into other relevant primary cells of the HIV-1 reservoir and SCID-Hu mouse models using the si143 mimetic target.

The potential gene therapy application of these siRNA mimetics is an exciting prospect. Mimetic constructs would be used in patients who have received effective cART and have residual viral reservoirs. TGS of HIV-1 by si/shRNA constructs, such as si/shRNA143 and/or PromA, delivered in retrovirally transduced autologous CD4^+^ T cells or CD34^+^ cells could be used to maintain reservoir silencing by enforcing latency in the absence of cART. However, extensive preclinical evaluation for evidence of *in vivo* efficacy, toxicity profiles, and delivery optimization is required before constructs move to clinic. The most tractable approach appears to be in humanized mouse models, which allow studies in primary CD4^+^ T cells and/or CD34^+^ stem cells, as performed for siRNA PromA.^22^ Multiplexing TGS-inducing siRNA mimetics may enhance the desired silencing effect, increasing breadth of strain coverage and reinforcing the latent-like state. Clearly, efficient delivery of multiplexed TGS-inducing siRNAs requires substantial evaluation and optimization. While the ultimate goal of viral eradication is commendable, an alternate functional cure where individuals continue to harbor the latent reservoir, but no longer require cART, is a novel approach to this formidable challenge.

## Materials and methods

*RNA duplexes.* Double-stranded RNA duplexes 19 bp in length with a 3′TT overhang were designed to target the HIV-1 5′LTR region and synthesized (Invitrogen, Scoresby, Australia). Sequences and locations within the HIV-1 5′LTR region are depicted in **Figure 1a**,**b** and listed in **Supplementary Table S1**.

*Cell culture.* 293T, MAGIC-5, HeLa T4+, and the HeLa CMV-3LTR1-4 cells were grown in DMEM supplemented with 10% FCS, 5 U/ml penicillin, and 50 mg/ml streptomycin (Gibco, Scoresby, Australia). HeLa T4+ and HeLa CMV-3LTR1-4 cells were maintained under selection with G418 (500 μg/ml) and Hygromycin (300 μg/ml), respectively (Gibco).

J-Lat 9.2 cells were grown in RPMI 1640 supplemented with 10% FCS, 5 U/ml penicillin, and 50 mg/ml streptomycin. J-Lat 9.2 cells were obtained through the NIH AIDS Reagent Program, Division of AIDS, NIAID, NIH: J-Lat Full Length Clone (clone 9.2) from Dr Eric Verdin.^52^ J-Lat 9.2 cells stably transduced with lentivirus vectors carrying shPromA or sh143 were grown under selection with Hygromycin (400 μg/ml) and Puromycin (1 μg/ml), respectively.

*HIV-1 GP-pseudotyped lentivirus generation, infection, and siRNA transfection.* HIV-1 pNL4.3Δenv containing a gag-GFP fusion reporter (HIV iGFP), VSV-G envelope, and psPAX2 plasmids were used to generate pseudotyped lentivirus vectors^61,62,63^ and HIV viral stocks tittered as described.^64^ RT activity of HIV-1 GFP pseudotyped lentivirus was determined to be 2,250 pg/μl as described.^65^ VSV-G pseudotyped lentivirus (TCID_50_ of 5 × 10^4^ − 50 μl) was added to 1 × 10^5^ pre-seeded 293T cells for 6 hours, and cultures were transfected with 100 pmol of each siRNA from the panel (**Figure 1**, **Supplementary Table S1**). Transfected cultures were incubated for 48 hours and analyzed for GFP expression using flow cytometry. Transfection of 293T, MAGIC-5, and HeLa T4+ cells used for 3′PTGS studies was performed using Lipofectamine 2000 (Invitrogen). All other transfections were performed using RNAiMax (Invitrogen).

*Viral quantitation.* RT activity in culture supernatants was determined as described.^65^ HIV-1 mRNA was quantified using real-time RT-PCR assays specific for HIV-*gag* as described.^20^

*Assessment of PTGS induced by U3 region-targeted siRNAs.* A HeLa T4+ cell line stably expressing high levels of the HIV-1 3′-LTR region overlapping into the *nef* region, designated CMV-3LTR1-4 (ref. 19), was utilized to assess PTGS contribution to siRNA-induced silencing. Briefly, CMV-3LTR1-4 cells were transfected with the siRNA panel (100 pmol/l) and variants. Following 48 hours, cells were harvested and 3′LTR mRNA expression was measured by SYBR-Green based quantitative real-time PCR assays using the primer set NUAf, 5′-CCAAAGAAGACAAGATATCCTTGA-3 and Chips2r, 5′-GCAGCTGCTTATATGCAGCATCTG-3 as described.^19^ Two positive controls were included, siRNA Nef366^46^ and siRNA PolyA, which both induce PTGS. Data were normalized to a GAPDH control, and statistical comparisons were made between mock-transfected cultures and siRNA-transfected cultures.

*Drug treatments.* In order to investigate reactivation of virus, HeLa-T4+ cells were treated for 24 hours with the HDAC inhibitors TSA (50 nmol/l) and SAHA (2.5 µmol/l) (Sigma-Aldrich, Castle Hill, Australia) on day 8 following HIV-1 infection and siRNA-transfection. Positive controls included siRNA- and mock-transfected cultures treated with TNF (10 ng/ml) (Sigma-Aldrich) for 24 hours. DMSO was added to the mock cultures at the concentration used to reconstitute the lyophilized drugs. All cultures were harvested at the same time and analyzed for intracellular viral mRNA levels by RT-PCR as described above. Experiments investigating potential off-target effects of the novel candidate siRNAs included positive controls generated by treatment of HeLa-T4+ cells for 24 hours with IFNα (500 IU/ml) or treatment with Poly (I:C) (10 ng/ml for 6 hours) before harvesting the cells. J-Lat 9.2 latent cell model cultures were treated with various concentrations of SAHA (0–100 µmol/l), TNF (0–100 ng/ml), or a combination of SAHA and TNF (0–25 µmol/l, 0–100 ng/ml) for 48 hours. GFP expression was measured using flow cytometry (LSRII Fortessa-BD, North Ryde, Australia) to detect reactivation of integrated HIV-1 provirus and analyzed using FlowJo.

*ChIP assays.* HeLa T4+ cells (5 × 10^5^) were seeded and infected with HIV-1_SF162_ (1,000 pg of RT/μl), then at day 3 postinfection, cultures were transfected with 300 pmol of appropriate siRNAs. Cultures were harvested 48 hours posttransfection for ChIP analysis using the EZ Magna A/G ChIP Kit as per manufacturer's instructions (Merck Millipore, Bayswater, Australia). Briefly, pellets were sonicated for 20 minutes (1 minute off, 1 minute on) in a COVARIS 5 sonicator at 5% duty cycle: intensity 4, burst/cycles 200, and protein isolated according to manufacturer's instructions (Merck Millipore, Bayswater, Australia). Fragmented chromatin was immunoprecipitated using antibodies (5 µg/ml) specific to histones H3K27me3 (#17–622), H3K9me3 (#17–625), H3K9Ac (#17–658), Ago1 (#07-599), and Rabbit-IgG (#PP64B) obtained from Merck Millipore as described.^19,21^

*Real-time PCR detection of interferon stimulated gene expression.* The mRNA expression levels of five IFNα response genes, ISG20, Viperin, IFIT1, and OAS1 were detected by SYBR-Green–based quantitative real-time PCR assays, as previously described.^66^ The following primer sets were utilized: ISG20; Fwd 5′- TCG TTG CAG CCT CGT GAA C-3′ and Rev 5′- TCC CTC AGG CCG GAT GA-3′, Viperin; Fwd 5′-GTG AGC AAT GGA AGC CTG ATC-3' and Rev 5′-GCT GTC ACA GGA GAT AGC GAG AA-3′, IFIT1; Fwd 5′-AAC TTA ATG CAG GAA GAA CAT GAC AA-3′ and Rev 5′-CTG CCA GTC TGC CCA TGT G-3′, and OAS1; Fwd 5′- AGG TGG TAA AGG GTG GCT CC -3′ and Rev 5′- ACA ACC AGG TCA GCG TCA GAT -3′. ISG20, Viperin, IFIT1, and OAS1 have all been used to detect the downstream effects of the IFNα response induced by RNA duplexes as previously reported.^36,37^

*Phosphorylated PKR immunoblotting.* Phosphorylated dsRNA-dependent protein kinase R (P-PKR) was detected as previously described.^67^ Briefly, cells were washed twice with PBS and lysed in cold lysis buffer (Reporter Gene Assay Lysis Buffer, Roche Diagnostics, Castle Hill, Australia) with protease inhibitor cocktail and phosphatase inhibitors (Roche Diagnostics). Cell extract was resolved by SDS–PAGE using an Invitrogen NuPage 4–12% Bis–Tris gel, which was separated for 35 minutes at 200 V. Separated proteins were transferred onto a polyvinylidene difluoride membrane for 90 minutes at 30 V, blocked in 2.5% skimmed milk/TBS and immunoblotted with mouse anti-β-actin (1:2,500; Sigma-Aldrich; A5441 clone AC-15) or rabbit polyclonal antiphosphorylated PKR (1:1,000; BioSource; Invitrogen, Scoresby, Australia, (pT451)) at room temperature for 1 hour. All dilutions were prepared in 2.5% skimmed milk/TBS. Prior to addition of secondary sheep anti-mouse HRP antibody or goat anti-rabbit HRP antibody used at 1:5,000 dilutions, respectively. Immunoblots were washed in TBS/0.1% Triton X-100, developed using Immuno-Star HRP chemiluminescent reagents as per manufacturer's instructions (Bio-Rad, Gladesville, Australia) and visualized using SynGene G:Box chemiluminescence software.

*Generation of stably transduced J-Lat 9.2 cell lines with shRNA targets.* The J-Lat 9.2 cell model of HIV-1 latency contains full-length latent HIV provirus with GFP substituted in the place of *nef* to allow detection of transcriptional activation of latent provirus.^52^ We transduced J-Lat 9.2 cells with lentiviral vectors containing the reporter mCherry (GeneCopoeia, Rockville, MD) and our shRNA of interest; sh143, shPromA, or dual transduced sh143 and shPromA and maintained selection of transduced cells with Puromycin (1 μg/ml), Hygromycin (300 μg/ml), or a combination of both antibiotics. Following ~2–3 weeks of selection, cells were sorted based on mCherry expression to obtain purified J-Lat 9.2 cell lines stably expressing sh143, shPromA, or dual sh143/shPromA. Parental J-Lat 9.2 cells were used as a control in reactivation experiments.

*Transduction of MDMs with shPromA and HIV-1JRFL infection.* Monocytes were purified from peripheral blood mononuclear cell using positive selection with anti-CD14 magnetic beads as per the manufacturer's protocol (Miltenyi Biotech, Gladbach, Germany), except for using 4°C. MDMs were generated by plastic adherence of monocytes in serum-free media for 1 hour prior to supplementation with RPMI 1640 media containing 10% human AB serum (Gibco). Following 7 days of differentiation, MDMs were transduced with lentivirus shPromA-JRFL or the specificity control shPromA-M2 or mock-transduced as described.^22^ MDM cultures were incubated with lentivirus for 5 days and then infected with HIV-1_JRFL_ (50 pg, RT equivalent) for 24 hours, then washed in PBS. RT activity and HIV-1 mRNA levels were analyzed 8 days postinfection as described.^22^

*Statistical analysis.* Pseudotyped virus data are shown as mean ± SD, RT values as mean ± SEM, and data sets were analyzed using a paired, two-tailed *t*-test. PTGS 3′LTR, drug reactivation, ChIP, off-target data, and transduced MDM data were analyzed using a nonparametric Mann–Whitney test and are shown as mean ± SEM. *P* < 0.05 was considered statistically significant. Analyses were performed using Prism Version 6.0 (Graphpad Software, San Diego, CA).

**SUPPLEMENTARY MATERIAL** **Table S1.** Novel candidate siRNA sequences targeting the HIV-1 5′LTR region.

## Supplementary Material

Supplementary Table S1 Novel candidate siRNA sequences targeting the HIV-1 5′LTR region.
