# Supplementary figures and images for: Novel RNA Duplex Locks HIV-1 in a Latent State via Chromatin-mediated Transcriptional Silencing
Source: Mol Ther Nucleic Acids. 2015 Oct 27;4(10):e261–. doi: 10.1038/mtna.2015.31 (PMC4881759; doi:10.1038/mtna.2015.31)

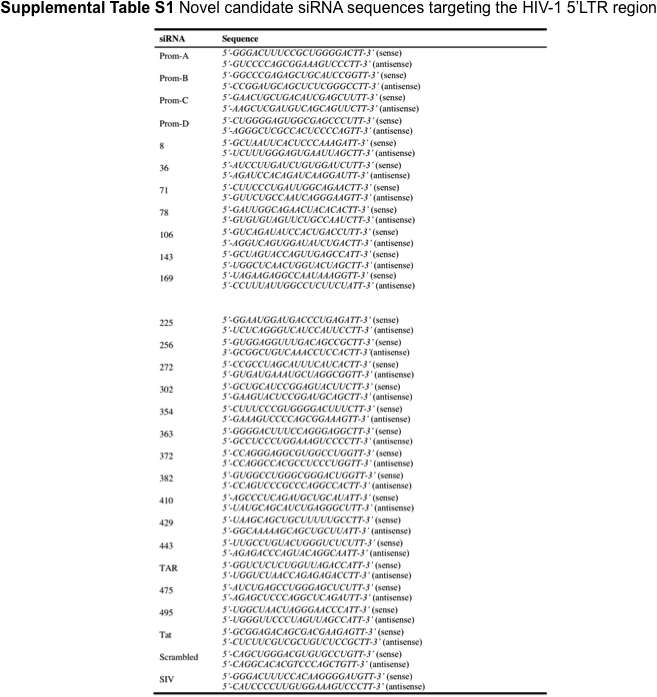

Supplement: Supplementary Table S1 — Novel candidate siRNA sequences targeting the HIV-1 5′LTR region. [file mtna201531x1.tiff]
